# The Effect of Spinal Needle Type on Post-Dural Puncture Headache in Spinal Anesthesia: Prospective Randomized Study

**DOI:** 10.5152/eurasianjmed.2024.23223

**Published:** 2024-02-01

**Authors:** Duygu Akyol, Mine Çelik, Necmiye Ay, Güneş Özlem Yıldız

**Affiliations:** 1 Clinic of Anesthesiology and Reanimation, Başakşehir Çam and Sakura City Hospital, Istanbul, Turkey; 2 Clinic of Anesthesiology and Reanimation, Istanbul Haseki Training and Research Hospital, Istanbul, Turkey; 3 Clinic of Anesthesiology and Reanimation, University of Health Sciences, Istanbul Bakirkoy Dr. Sadi Konuk Training and Research Hospital, Istanbul, Turkey

**Keywords:** Cesarean section, obstetric anesthesia, spinal anesthesia, spinal needle type

## Abstract

**Background:**

Postdural puncture headache is a headache that occurs after a dura puncture, especially in caesarean sections, and affects patient comfort and mobilization. In this study, we compared the effects of pencil-tipped spinal needles and especially curved, bilateral atraumatic spinal needles in individuals undergoing elective caesarean sections.

**Methods:**

A total of 886 patients, aged 20–50 years, who had cesarean sections with spinal anesthesia and had American Society of Anesthesiologists II and III scores, were included in the study. The patients were allocated into 3 groups using the closed envelope randomization technique: Group 1 (n = 250) received spinal insertions using 25-gauge pencil-point needles; Group 2 (n = 245) received spinal insertions using 26-gauge atraumatic needles; and Group 3 (n = 250) received spinal insertions using 27-gauge pencil-point needles. Records were kept of the quantity of spinal needle referrals, the type of treatment, the length of hospital stays, and complications.

**Results:**

In the study, 745 patients who had cesarean section operations under spinal anesthesia were further analyzed. The mean incidence of post-dural puncture headache (PDPH) was 3.2% (n = 24). The incidence of PDPH was higher in group 2 than in group 3 and group 1 (Group 1: 2.8%; Group 2: 6.8%; Group 3: 0%) (*P* < 0.05). Among other complications, low back, back, shoulder, and surgical complications were similar for all 3 groups.

**Conclusion:**

In caesarean section operations, pencil-point spinal needles were found to have a lower incidence of postdural puncture headache than Atraucan-cut needles, regardless of needle thickness.

**Main Points**

- The incidence of post-dural puncture headache (PDPH) after spinal anesthesia increases in women, especially in cesarean section.
- The incidence of PDPH should be reduced in order to increase patient comfort and strengthen the mother-baby bond.
- Apart from many factors, spinal needle type and thickness are also important in the incidence of PDPH.
- In order to reduce the incidence of PDPH, the use of pencil-point spinal needles should be increased compared to atraumatic needles.

## Introduction

Spinal anesthesia, which is currently preferred because it is safer than general anesthesia and has a lower complication rate, also has advantages such as lower cost and easier patient management.^1^ Although the complication rate is low, post-dural puncture headache (PDPH) is the most typical.^2^ According to the International Classification of Headache Disorders criteria, PDPH is defined as a type of headache that occurs within 5 days following dural puncture, increases when standing up, relieves when lying on the back, and may be accompanied by tinnitus, photophobia, and nausea.^3^ Especially in pregnant women, increased estrogen levels increase the risk of PDPH by affecting cerebral vascular tone and decreasing cerebrospinal fluid pressure.^4^ Post-dural puncture headache due to spinal anesthesia is an important cause of maternal morbidity in the postpartum period, leading to prolongation of the discharge time, increased hospitalization and costs.

Post-dural puncture headaches may develop due to patient-related or technical reasons,^3,5^ and they can be prevented or minimized by correcting some controllable factors.^6^ Patient-related causes include gender and age. Among the technical reasons, needle type, needle thickness, number of trials, and experience can be counted. Many types of spinal needles have been described in spinal anesthesia practice and are basically classified as sharp and blunt-tipped. Blunt-tipped needles are sprotte, whitacre (pencil-point), and grain needles, and the cutting tips are Quincke-babcock, Atraucan, and Pitkin needles.^7^ There is no common approach in the literature regarding which needle type is more advantageous. Many clinics have different individual applications.

In this study, our primary aim was to evaluate the relationship between needle type and PDPH. Our secondary aim was to evaluate the number of spinal needle attempts, length of hospital stay, and complications.

## Material and Methods

### Compliance with Ethical Standards

Following the Ethics committee of Başakşehir Çam and Sakura City Hospital approval (dated December 16, 2021, approval number: 2021-97), the study was started, and it was planned in compliance with the tenets of the Helsinki Declaration. All patients provided written informed consent prior to surgery and research participation. Clinicals trial registration (protocol ıd: 2021-297, NCT05777694) was included in our study.

### Study design

**Figure 1.**
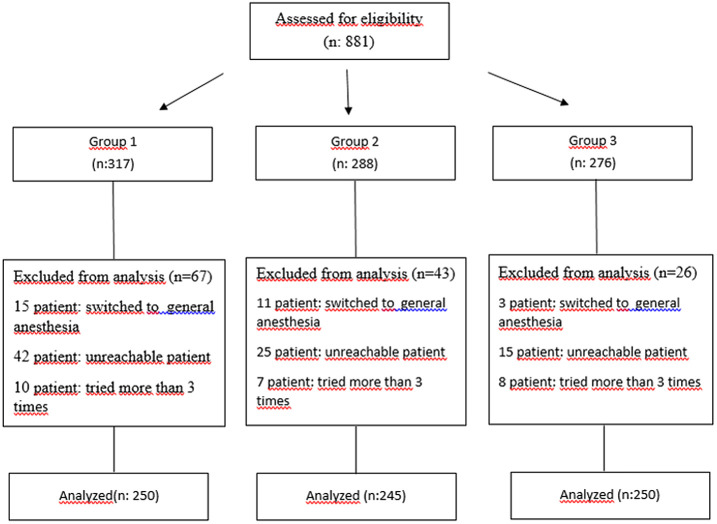
Study flow diagram.

Between February 2022 and May 2022, a total of 881 patients, aged 20–50 years, who had cesarean sections with regional anesthesia and had American Society of Anesthesiologists (ASA) II and III scores, were included in the study. A sample size of 242 achieves 80% power to detect an effect size (W) of 0.2 using a 2 degrees of freedom Chi-Square Test with a significance level (alpha) of 0.05. The patients were divided into 3 groups using the closed envelope method, with Group 1 (n = 250) receiving a 25-gauge pencil-point spinal needle (Pencan® 0.53 88 mm-G25 312, B. Braun, Melsungen, Germany), Group 2 (n = 245) receiving a 26-gauge atraumatic spinal needle (Atraucan® 0.47 88 mm-G26 312, B. Braun, Melsungen, Germany), and Group 3 (n = 250) receiving a 27-gauge pencil-point spinal needle (Pencan® 0.47 88 mm-G27 312, B. Braun, Melsungen, Germany) (Figure 1). The exclusion criteria were failure of regional anesthesia, contraindication to regional anesthesia, a mass index (BMI) of 50 kg/m² and above, and conversion to general anesthesia due to reasons such as intraoperative bleeding and hemodynamic changes.

### Regional Anesthesia Technique

A fasting period of 6-8 hours was applied in all patients. Patients were positioned supine with a left lateral displacement of 15-20 degree by putting a wedge under the right hip. Continuous 3-lead electrocardiograms, pulse oximetry, and non-invasive blood pressure monitoring were performed in accordance with ASA guidelines. Before spinal anesthesia, each patient received 15-20 mL/kg of crystalloid fluid solution over the course of 20 minutes through an 18-G intravenous cannula. Perioperative crystalloid infusion continued. The patients did not receive the pharmacological premedication. The anesthesiologists who performed the needle insertion have at least 5 years of expertise. Aseptic procedures were used to provide spinal anesthesia. After the patients with stable hemodynamics were placed in a sitting position, 10-15 mg of hyperbaric bupivacaine 0.5% (Bucain 0.5% hyperbaric®, Delta Select, Dreieich, Germany) and 20 µg of fentanyl were administered by the median approach from the L4-5 spinal space following sight of transparent cerebrospinal fluid (CSF) flow. Then, the patient was placed in the supine position, and oxygen support was provided at 5 L/min with a face mask. The sensory block level was evaluated every 5 minutes by the loss of the pinprick sensation. The Bromage scale (0 = free movement of legs and feet; 1 = just able to flex knees with free movement of feet; 2 = unable to flex knees but with free movement of feet; 3 = unable to move legs or feet) was used to evaluate the motor block of the lower extremities. After adequate motor and sensory blockage, the operation stage was started by the gynecology and obstetrics specialist. Hypotension was defined as a 20% drop in systolic blood pressure and/or a blood pressure reading below 90 mm Hg. Patients who experienced hypotension received 250 mL of IV bolus crystalloid fluid and 5-10 mg of IV ephedrine. Colloid intravenous infusion was started in patients with perioperative bleeding and hypotension despite crystalloid infusion and ephedrine. Those with a heart rate below 50/min were considered to have bradycardia, and 0.01 mg/kg of IV atropine was administered.

### Post-Operative Follow-up

Patients were observed in the postoperative period. Kept for 1-2 hours in the anesthesia care room. Intravenous paracetamol (3 × 1 g) and intramuscular diclofenac sodium (2 × 75 mg) were routinely administered to the patients during ward follow-up. For 6 hours, the patients were confined to bed. Patients received 2-3 liters of hydration every day, and anesthesiologists questioned patients about headaches, back discomfort, and other problems (tinnitus, photophobia, nausea, etc). Patients were visited at regular intervals from postoperative hospitalization until discharge. After discharge, the presence of postspinal headache was evaluated by calling on the first, seventh, and fifteenth postoperative days.

Bed rest in the supine position was applied to the patients diagnosed with PDPH during hospitalization. Intravenous crystalloid, theophylline, and perioral caffeinated nonsteroidal anti-inflammatory drugs were added to the treatment.

In the telephone interviews after hospital discharge, patients diagnosed with PDPH were advised to increase perioral fluid intake and bed rest, and perioral caffeinated nonsteroidal anti-inflammatory drugs were added to the treatment.

Epidural blood patch application was planned for patients with severe spinal headache despite conservative and medical treatment.

### Statistical Analysis

Power analysis: A sample size of 242 achieves 80% power to detect an effect size (W) of 0.2 using a 2 degrees of freedom Chi-Square Test with a significance level (alpha) of 0.05.

Data were analyzed using Statistical Package for Social Science Statistics software for Windows, version 20 (IBM SPSS Corp.; Armonk, NY, USA). The normal distribution of the data was evaluated using the Kolmogorov–Smirnov test. The normally distributed variables were presented as the mean ± standard deviation, while the non-normally distributed variables were presented as the median (interquartile range: 25%-75%). Categorical variables were presented as numbers and percentages. The analysis of variance test (post-hoc: Bonferroni correction) was used for the group comparison of the normally distributed variables, and the Kruskal–Wallis *H* test (post-hoc: Dunn’s correction) was used for the intergroup comparison of the non-normally distributed variables. The chi-square and Fisher exact tests were used for the intergroup comparison of the categorical variables. The *P *< .05 was accepted as statistically significant.

## Results

In the study, 881 patients who had cesarean section operations under spinal anesthesia were included. General anesthesia was used in patients with 4 or more trials, insufficient sensory block, impaired hemodynamics, severe hypotension, respiratory distress, and significant bleeding. The study removed a total of 136 individuals, including 67 from group 1, 43 from group 2, and 26 from group 3. The additional analysis included 745 patients in all. In none of the patients was an epidural blood patch necessary.

The demographic data of the patients age, weight, BMI, ASA, operation time, and hospitalization time did not differ statistically. The patients in groups 1, 2, and 3 had respective mean ages of 29.9 ± 5.2, 30.2 ± 5.7, and 30.2 ± 5.6. The ASA III rates between the groups were 2.4%, 3.3%, and 3.2%, respectively. The number of spinal trials in all 3 groups was not statistically significant (Table 1).

**Figure 2.**
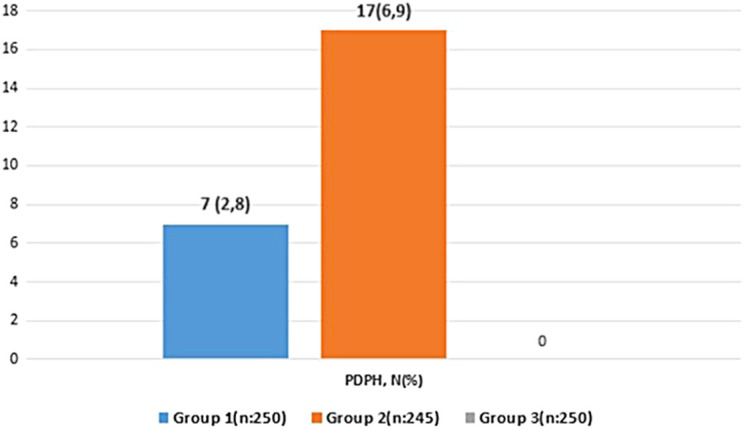
Incidence of Post-Dural Puncture HeadachePDPH.

The mean incidence of PDPH was 0.03% (n = 24). PDPH was statistically significant when evaluated between the 3 groups. The incidence of PDPH was higher in group 2 than in group 3 and group 1 (Group 1: 2.8%; Group 2: 6.9%; Group 3: 0%) (*P *< .05) (Figure 2). Among other complications, low back, back, shoulder pain, and surgical complications were similar for all 3 groups (Table 2).

## Discussion

In our study, we evaluated the effect of the same brand of Atraucan and pencil-point spinal needles on the incidence of PDPH. The 26-gauge Atraucan atraumatic spinal needles had a higher incidence of PDPH than 25- and 27- pencil-point spinal needles. The number of spinal needle procedures, anaesthetics, or surgical complications were similar in the 3 groups. All patients responded to conservative and medical treatment for PDPH. An epidural blood patch was not required in any patient.

The incidence of PDPH after spinal anesthesia is in a wide range.^8-10^ Pirbudak et al reported 10.8% PDPH after cesarean section in their study.^11^ In other research, the prevalence of PDPH following spinal anesthesia was discovered to range from 6% to 36%.^8,10,12^ In our study, the incidence of PDPH was 0.03%. The incidence of PDPH was statistically higher in the group using 26 g of Atraucan.

In a meta-analysis, pencil-point spinal needles are thought to reduce the prevalence of PDPH compared to quinke-type spinal needles because they limit dural fiber damage and CSF loss.^13^ In many studies, it has been shown that 25 g Whitacre pencil-point spinal needles used in caesarean sections reduce the incidence of PDPH compared to 25 g quinke-type spinal needles.^14-16^ A pencil-point spinal needle is recommended by the American Academy of Neurology. Therefore, the use of quinke-type spinal needles has decreased.^17^

The importance of needle size in the development of PDPH has been proven.^16,18^ However, as the thickness of the spinal needle becomes thinner, it increases the number of spinal attempts, increasing the risk of dural puncture and thus the incidence of PDPH.^13^ Despite this, no correlation could be found between the incidence of PDPH and different-size pencil-point spinal needles applied in obstetric patients.^19,20^ However, the impact of needle thickness on PDPH was not proven in our investigation. One of the modifiable risk factors for the occurrence of PDPH, in addition to spinal needle size, is the shape of the spinal needle. It has also been shown that the Atraucan spinal needle has less bos leakage than the sharp or pencil-point needle, and that the sharp and pencil-point spinal needle have the characteristics of both.^21^ With 26-gauge Atraucan and pencil-point spinal needles, PDPH incidence was comparable in obstetric patients. The Atraucan group, however, required more epidural blood patches on average.^22^ In this study, which evaluated randomized controlled studies performed in many centers, it was observed that traumatic needles increased the incidence of PDPH almost twice compared to atraumatic needles.^23^ The incidence of PDPH was discovered to be comparable in another investigation comparing blunt-tipped spinal needles to 26 G (Atraucan) spinal needles.^24^ In the study of Batova et al, it was shown that an atraumatic needle does not reduce PDPH.^25^

Position, fluid therapy, drug therapy, and an epidural blood patch can be used in the treatment of PDPH.^26,27^ In patients with PDPH, it has been shown that intravenous caffeine, sodium benzoate, and oral caffeine, and even combined analgesics such as caffeine and acetaminophen, reduce pain.^28^ Our study is also supportive, as all patients with PDPH respond to treatment.

Our study was single-center, which is its principal drawback.

In our study, a higher incidence of PDPH was observed in Atraucan atraumatic needles because they have sharp needle properties as well as pencil-tipped needle properties. Therefore, the use of pencil-point spinal needles in obstetric anesthesia may be recommended to reduce the incidence of PDPH.

## Figures and Tables

**Table 1.** Demographic and Clinical Data of Patients

	Group 1 (n : 250)	Group 2 (n : 245)	Group 3 (n : 250)	*P*
Age	29.9 ± 5.2	30.2 ± 5.7	30.2 ± 5.6	.7
Weight, kg	80 ± 13.4	79.4 ± 13.4	79 ± 12.7	.7
BMI, kg/m^2^3	30.7 ± 5.5	31 ± 8.5	30.5 ± 4.9	.6
Operation time, minute	64.8 ± 11.8	67 ± 10.6	66.9 ± 15.1	.08
ASA II/ III	246 (97.6)/6 (2.4)	237 (96.7)/8 (3.3)	242 (96.8)/8 (3.2)	.8
Comorbidity	3			.09
HT, n (%)	4	7	8	3
DM, n (%)	9	9	14	
Asthma, n (%)	3	6	1	
Thyroid disorders, n (%)	14	14	21	
Others, n (%)	7	3	9	
Length of stay in hospital, day	2.2 ± 0.7	2.2 ± 0.7	2.1 ± 0.8	.3
Needle attempts	1.2 ± 0.46	1.2 ± 0.5	1.3 ± 0.5	.06

Numerical variables with normal distribution were shown as mean ± standard deviation. Numerical variables that do not show normal distribution are shown as median (IQR). Categorical variables were shown as numbers (%). ASA, American Society of Anesthesiologists; BMI, Body Mass Index; DM, Diabetes Mellitus; HT, Hypertension.

**Table 2.** Non-Post-Dural Puncture Head Anesthesia Complications and Surgical Complications

	Group 1 (n : 250)	Group 2 (n : 245)	Group 3 (n : 250)	*P*
Non-surgical complications	3	3	3	.53
Backache, n (%)	21 (8.4)	13	24	3
Shoulder pain, n (%)	27 (10.8)	24	22	
Back pain, n (%)	1 (0.4)	0	0	
Surgical complications, n (%)	17 (6.8)	7	11	.11
